# The charcoal trap: Miombo forests and the energy needs of people

**DOI:** 10.1186/1750-0680-6-5

**Published:** 2011-08-19

**Authors:** Werner L Kutsch, Lutz Merbold, Waldemar Ziegler, Mukufute M Mukelabai, Maurice Muchinda, Olaf Kolle, Robert J Scholes

**Affiliations:** 1 Johann Heinrich von Thünen Institute (vTI), Institute for Agricultural Climate Research, Bundesallee 50, 38116 Braunschweig, Germany; 2 ETH Zurich, Institute of Agricultural Sciences (IAS), Grassland Sciences Group, Universitätstrasse 2, 8092 Zurich, Switzerland; 3 Max-Planck Institute for Biogeochemistry, P.O Box 100164, 07701 Jena, Germany; 4 Zambia Meteorological Department, Haile Sellasie Avenue, City Airport, P. O. Box 30200, 10101 Lusaka, Zambia; 5 CSIR Natural Resources and Environment, Box 395, Pretoria 0001, South Africa

**Keywords:** *miombo *woodland, deforestation, biomass, eddy covariance, soil carbon

## Abstract

**Background:**

This study evaluates the carbon dioxide and other greenhouse gas fluxes to the atmosphere resulting from charcoal production in Zambia. It combines new biomass and flux data from a study, that was conducted in a *miombo *woodland within the Kataba Forest Reserve in the Western Province of Zambia, with data from other studies.

**Results:**

The measurements at Kataba compared protected area (3 plots) with a highly disturbed plot outside the forest reserve and showed considerably reduced biomass after logging for charcoal production. The average aboveground biomass content of the reserve (Plots 2-4) was around 150 t ha^-1^, while the disturbed plot only contained 24 t ha^-1^. Soil carbon was not reduced significantly in the disturbed plot. Two years of eddy covariance measurements resulted in net ecosystem exchange values of -17 ± 31 g C m^-2 ^y^-1^, in the first and 90 ± 16 g C m^-2 ^in the second year. Thus, on the basis of these two years of measurement, there is no evidence that the *miombo *woodland at Kataba represents a present-day carbon sink. At the country level, it is likely that deforestation for charcoal production currently leads to a per capita emission rate of 2 - 3 t CO_2 _y^-1^. This is due to poor forest regeneration, although the resilience of *miombo *woodlands is high. Better post-harvest management could change this situation.

**Conclusions:**

We argue that protection of *miombo *woodlands has to account for the energy demands of the population. The production at national scale that we estimated converts into 10,000 - 15,000 GWh y^-1 ^of energy in the charcoal. The term "Charcoal Trap" we introduce, describes the fact that this energy supply has to be substituted when woodlands are protected. One possible solution, a shift in energy supply from charcoal to electricity, would reduce the pressure of forests but requires high investments into grid and power generation. Since Zambia currently cannot generate this money by itself, the country will remain locked in the charcoal trap such as many other of its African neighbours. The question arises whether and how money and technology transfer to increase regenerative electrical power generation should become part of a post-Kyoto process. Furthermore, better inventory data are urgently required to improve knowledge about the current state of the woodland usage and recovery. Net greenhouse gas emissions could be reduced substantially by improving the post-harvest management, charcoal production technology and/or providing alternative energy supply.

## Background

*Miombo *woodlands cover the transition zone between the dry open savannas and moist forests in Southern Africa [1]. Being located in sub-humid areas with a distinct dry season and having a discontinuous tree cover, they belong to the broad concept of savannas [2]. Mean annual precipitation (MAP) is commonly above 600 mm and tree cover exceeds 40%, a threshold that separates the open savannas from the more closed woodlands. Where MAP exceeds 800 mm tree cover may be larger than 60%, the threshold for forests. *Miombo *woodlands occupy up to 2.7 million km^2 ^in Southern Africa and provide many ecosystem services supporting rural life. The specific ecosystems provide medical products, wild foods such as mushroom and certain roots, timber for construction and most important fuel [3].

In all Southern African countries, Zambia given as a case study, these woodlands are experiencing accelerating degradation and clearing, through the production of charcoal being the initial driver. Domestic energy needs in the growing urban areas are largely satisfied by charcoal, which is less an energy-efficient fuel on a tree-to-table basis than the firewood commonly used in rural areas. However, having a higher energy density than firewood charcoal is cheaper to transport. The commercialisation of charcoal production for supplying the urban demand has changed the spatial pattern of deforestation. Whereas in rural areas firewood is mostly harvested in small amounts as dead wood by the consuming households themselves, charcoal is now produced in large quantities by felling live trees in the vicinity of a makeshift kiln, and is traded over much longer distances [4,5].

As a consequence, charcoal production has become a full-time job for migrant workers. These workers 'buy' the trees from local communities, produce the charcoal and leave. The price per tree in 2009 in the Western Province of Zambia was reported to be about 1000 ZMK (less than 20 Eurocent). Migrant charcoal producers are not bound to the land and the ecosystem services the *miombo *woodlands provide and have a less intentions to use of the ecosystem sustainably. Over the last decade, charcoal production has become non-sustainable in many areas, meaning that the fraction of the landscape cleared annually exceeds the fraction that is enabled to re-grow [5,6]. However, any strategy to reduce the pressure on woodlands has to take aspects of energy supply into account. It is only likely to succeed, if accompanied by a strategy that shifts domestic energy consumption to non-woodland sources. Otherwise, the country will continue to be locked in what we want to call 'the charcoal trap': due to missing alternatives in energy supply and employment, non-sustainable charcoal production in *miombo *woodlands will continue until the forest will be disappeared completely.

This study used data from inventories and from eddy covariance measurements of carbon exchange to characterize the impact of charcoal production on *miombo *woodlands. We address the following questions: **(i) **how much carbon is lost at local scale and **(ii) **does forest degradation result in the loss of a carbon sink? On the basis of our data and additional data we **(iii) **estimate the per capita emissions through deforestation and forest degradation in Zambia and relate it to fossil fuel emissions. Furthermore, **(iv) **a rough estimate of the energy that is provided by charcoal production to private households at a national level is calculated and **(v) **options for alternative energy supply to private households are discussed. We are aware that the results we present are very uncertain due to the very small database that is available. However, our approach may show how important it is to bridge the gap between biogeochemical studies and socio-economic aspects as well as local and national policies on forest ecosystem management. Therefore, we see our study also as a methodological prototype that points out where future data have to be obtained and that could be reproduced in other countries of Sub-Saharan Africa.

## Results

### Carbon loss and energy gain at site level

**Figure 1:**
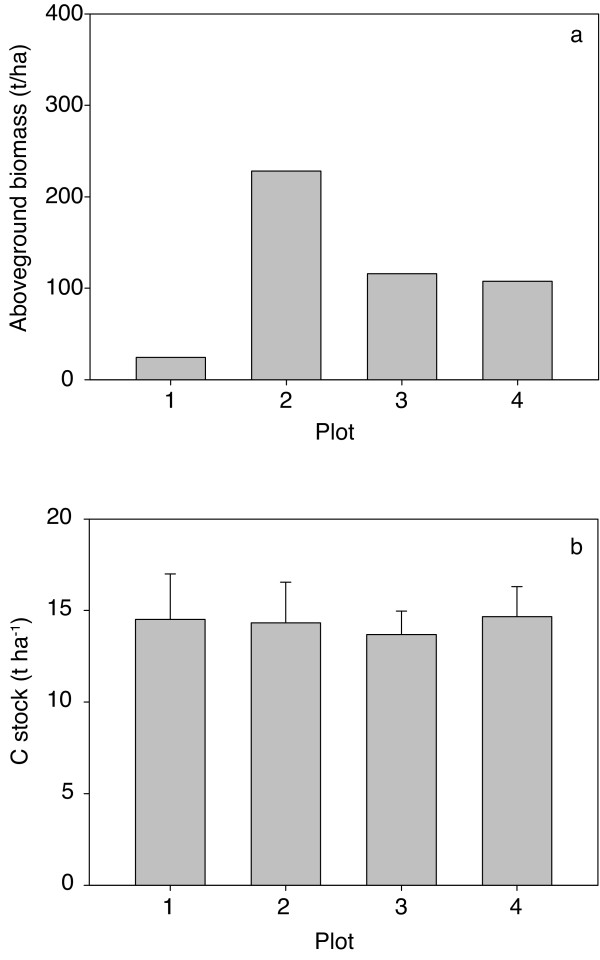
**Graphical presentation of plots**. a: Aboveground biomass of the four inventory plots. b: Soil carbon stocks in the upper 30 cm of the four inventory plots.

Values of aboveground and belowground carbon are compiled in Figure 1 and Tables 1 and 2. They show high variances in aboveground biomass between and within the plots in the forest reserve as well as considerably reduced biomass after logging. The largest aboveground biomass was detected in Plot 2, where fewer but bigger trees were located (Figure 1a). This plot was slightly disturbed by the logging of few single trees. The average aboveground biomass content of the reserve (Plots 2-4) was around 150 t ha^-1 ^while the disturbed plot only contained 24 t ha^-1^: thus the loss by charcoal production was 126 t ha^-1 ^This converts into 284 GJ ha^-1 ^of energy as charcoal when Equation 2 is applied. Soil carbon was not reduced significantly in the disturbed plot (Figure 1b and Table 2).

**Table 1:** Aboveground biomass in the inventory plots

Plot	Aboveground biomass (t ha^-1^)	Variance	Number of trees	Av. height (m)	Av. DBH (cm)
1	24.49	42.17	53.00	5.54	8.88
2	228.18	2158.12	121.00	7.72	14.29
3	115.95	54891.41	277.00	5.75	9.34
4	107.55	39.17	488.00	7.61	7.45

**Table 2:** Soil carbon stocks in the inventory plots

Plot	C stock t ha^-1 ^(0 - 30 cm)	SE	variance	***n***
1	14.51	2.50	50.16	9
2	14.33	2.23	59.73	13
3	13.70	1.26	28.27	19
4	14.66	1.65	27.35	11

### Is a carbon sink lost by forest degradation?

**Figure 2:**
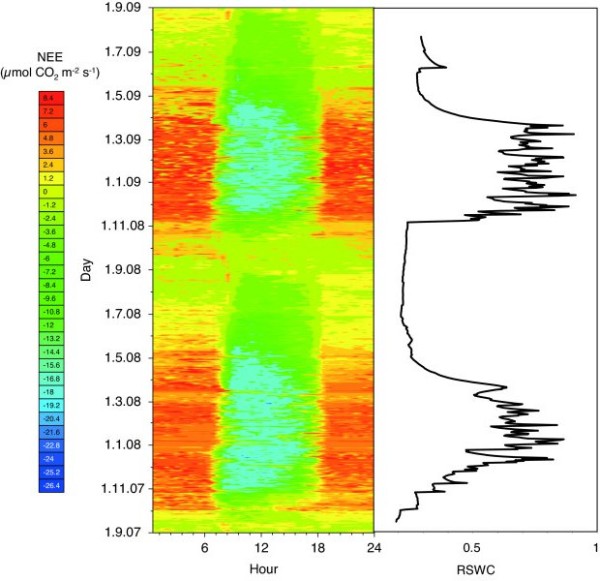
**Fingerprint of two years of CO_2 _exchange between the Kataba Forest reserve and the atmosphere and relative soil water content down to a depth of 1 m**.

The net ecosystem exchange at Kataba, as measured by eddy covariance, is shown in Figure 2. It shows how strongly the *miombo *ecosystem is driven by water availability. The first rains in October result in an immediate increase in night-time respiration, followed by high daytime uptake rates from November on. During the peak growing season (Dec. - Feb.) the maximum net uptake rates were around -20 μmol CO_2 _m^-2 ^s^-1^, where by convention the negative sign represents a carbon sink. The net ecosystem exchange slowly returned to zero, and then became weakly positive (a carbon source) after the rains stopped in April.

Integrated over all the seasons, the NEE for the first year of our measurements (September 2007 to August 2008) was -17 ± 31 g C m^-2 ^y^-1^, in other words, statistically indistinguishable from zero. In the second year of measurement the woodland was a net carbon source of 90 ± 16 g C m^-2^. Thus, on the basis of these two years of measurement, there is no evidence that the *miombo *woodland at Kataba represents a present-day carbon sink.

### Up-scaling to country-wide rates

The country-wide deforestation rate in Zambia is highly uncertain. It was reported to be 445,000 ha y^-1 ^between 2000 and 2005 by an estimate of the FAO [6], 298,000 ha y^-1 ^by UN REDD [7], and only 166, 600 ha y^-1 ^by a more recent study of the FAO [5]. The reason for this wide range is a lack in a clear distinction between deforestation and forest degradation. The lowest number is commented by the authors of this report as followed: "There is a slight forest decrease, but the real problem is the forest degradation" [5]. The average of all studies available was 303,200 ha y^-1^. A similar uncertainty was found in the data about standing biomass. It ranged from 124 t ha^-1 ^in our study down to 43.2 t ha^-1 ^[6]. Since the value calculated from our study was quite high compared to all others we used the median instead of the average to calculate a mean value (61.7 t ha^-1^).

In Table 3 the different deforestation rates were combined with the different biomass data. On the basis of each data pair the whole country annual emissions from charcoal production were calculated (Table 3a). The range was from 13.2 Mt CO_2 _y^-1 ^to 102.8 Mt CO_2 _y^-1 ^with a mean value of 34.3 Mt CO_2 _y^-1^. Per capita emissions ranged from 1 to 8 t CO_2 _y^-1 ^with a mean of 2.7 t CO_2 _y^-1 ^(Table 3b).

**Table 3:** Total CO_2 _emissions (A), per capita CO_2 _emissions (B) and energy gained (C) from charcoal production in Zambia


**A) Total emissions (Mt CO_2 _y^-1^)**						
	Biomass →	This study	Chidumayo	UN REDD	FAO 2005	Median
↓ Deforestation rate		(126 t ha^-1^)	(70 t ha^-1^)	(53.3 t ha^-1^)	(43.2 t ha^-1^)	(61.7 t ha^-1^)
FAO 2005 (445 000 ha y^-1^)		102.8	57.1	43.5	35.2	*50.3*
UN REDD (298 000 ha y^-1^)		68.8	38.2	29.1	23.6	*33.7*
FAO 2010 (166 600 ha y^-1^)		38.5	21.4	16.3	13.2	*18.8*
Average (303 200 ha y^-1^)		*70.0*	*38.9*	*29.6*	*24.0*	**34.3**
						
						
**B) Per capita emissions (t CO_2 _y^-1^)**						
	Biomass →	This study	Chidumayo	UN REDD	FAO 2005	Median
↓ Deforestation rate		(126 t ha^-1^)	(70 t ha^-1^)	(53.3 t ha^-1^)	(43.2 t ha^-1^)	(61.7 t ha^-1^)
FAO 2005 (445 000 ha y^-1^)		8.0	4.4	3.4	2.7	3.9
UN REDD (298 000 ha y^-1^)		5.3	3.0	2.3	1.8	2.6
FAO 2010 (166 600 ha y^-1^)		3.0	1.7	1.3	1.0	1.5
Average (303 200 ha y^-1^)		5.4	3.0	2.3	1.9	**2.7**
						
						
**C) Gained energy (GWh y^-1^)**						
	Biomass →	This study	Chidumayo	UN REDD	FAO 2005	Median
↓ Deforestation rate		(126 t ha^-1^)	(70 t ha^-1^)	(53.3 t ha^-1^)	(43.2 t ha^-1^)	(61.7 t ha^-1^)
FAO 2005 (445 000 ha y^-1^)		35 047	19 470	14 825	12 016	*17 148*
UN REDD (298 000 ha y^-1^)		23 469	13 039	9 928	8 047	*11 483*
FAO 2010 (166 600 ha y^-1^)		13 121	7 289	5 550	4 499	*6 420*
Average (303 200 ha y^-1^)		*23 879*	*13 266*	*10 101*	*8 187*	**11 684**

The resulting charcoal energy provided to households in Zambia ranged between 4500 GWh y^-1 ^and 35,000 GWh y^-1 ^(Table 3c). The average estimate of the annual energy supplied by charcoal would be around 11,700 GWh y^-1^.

## Discussion

### Biomass losses, emissions and energy gain by charcoal production

Standing biomass in *miombo *woodlands is very variable at the scale of ten to hundred meters, as shown in Table 1 of this study but also by other studies [8-10]. Therefore it is difficult to derive a highly confident mean value for up-scaling and energy supply calculations. The average aboveground biomass in our study was about twice the average value given by Chidumayo [8] in his study on *miombo *woodland structure, but in the same range as his plot with highest biomass, which was located in a swath in the Lower Zambezi National Park resulting from woodland clearing for tsetse-fly control in 1972, and thereafter allowed to recover. Kataba Forest Reserve, where our study was conducted, was established in 1973 and previous land-use is unknown. Being undisturbed for at least 35 years it represents mature *miombo *woodlands and our inventory plots reflect the potential maximum biomass in *miombo *woodlands. The average is presumably in the range between 43 - 70 t ha^-1 ^[6,8,11]. This is confirmed by Ryan et al. [10], reporting 19 t C ha^-1 ^for stem biomass at a *miombo *site in Mozambique. UN REDD [7] showed that "choice of method has a large effect on the final carbon stock estimate. Depending on method, the above ground estimates span from approximately 15 t C ha^-1 ^to 39 t C ha^-1^" in their recent study. Assuming a carbon content of 45 to 50%, this represents a total aboveground biomass value within the presumed average range. The number we used to calculate the most probable emission rate (61.7 t biomass ha^-1^) was also within the range where most of the studies agree.

### The sink strength of *miombo *woodlands

During an observational period of two years the *miombo *woodland in Western Zambia revealed high annual rates of gross primary production (GPP) and total ecosystem respiration (Reco) but was not a significant carbon sink. GPP was higher than the mean GPP of deciduous forests recorded in temperate-humid or Mediterranean areas [12]. However, virtually all of the carbon fixed by photosynthesis was re-respired. The Reco/GPP ratio was 0.99 in the first year and 1.06 in the second year.

We postulate that the high ecosystem respiration is due to the age of the woodland, which has been undisturbed for several decades, and in the context of this fast-growing tropical forest is in its 'climax' state. The carbon allocation pattern in this ecosystem may be a contributing factor. Ecosystems based on nutrient-poor substrates are forced to allocate a large proportion of their assimilated carbon into nutrient capture [13,14]. In particular, mycorrhizas play an important role on sandy, low-phosphorus soils [15]. The Kataba *miombo *woodland is highly mycorrhizal: the dominant species have obligate ecto-mycorrhizal associations. Merbold et al. [16] showed that 80% of Reco at this site is attributable to soil respiration.

The magnitude and direction of carbon fluxes in this *miombo *woodland are highly dependent on soil water content, as also shown by Kutsch et al. [17] and Merbold et al. [18] using temporal and spatial analysis, respectively. Respiration responds to rain pulses much faster than photosynthesis does, a pattern recently described by Williams et al. [19] in a drier South African savanna ecosystem. In that system, Archibald et al. [20] came to the same conclusion (based on a longer temporal record) as we do at Kataba: annual NEE varies around the zero point, depending on the pattern of soil moisture during the year.

These findings do not support the results by Lewis et al. [21], who infer from tree inventories that carbon storage in live trees of undisturbed tropical rain forests in Africa is on average increasing by 63 g C m^-2 ^y^-1 ^- a phenomenon that has been attributed to changes in climate and rising CO_2_. Mueller-Landau [22] comments on the finding that many tropical forests are increasing in biomass, meaning forests may have been knocked from their previous equilibrium by global climate change and are in transition to a higher carbon state. Our results show that undisturbed deciduous woodlands in the semi-arid tropics may not be following the same dynamic, possibly because they are strongly limited by soil moisture and nutrients and thus benefit little from increased CO_2 _concentration. They also contrast recent model studies that predict strong carbon sinks in the moist savannas and woodlands of Southern Africa but are unconstrained by any *in-situ *observations [23].

Our study represents the only continuous measurement of net ecosystem exchange of CO_2 _in miombo woodlands. Previous measurement campaigns covered only short periods [24]. Although we covered two full growing seasons the study is of limited duration and at one single location only. Thus the carbon balance of the *miombo *woodlands as a whole remains an open question. Assuming that our study site with its high aboveground biomass reflects the upper threshold of potential carbon storage, the neutral fluxes seem to be logical. Younger or disturbed woodlands are likely to continuously sequester carbon during re-growth of the trees. Williams et al. [9] reported from inventories at re-growing woodlands a carbon sink of 0.7 t C ha^-1 ^y^-1 ^(70 g C m^-2 ^y^-1^) We can conclude that there is no empirical evidence that deforestation or degradation of old-growth *miombo *woodlands will result in the loss of a significant carbon sink but may initiate a carbon sink during re-growth.

### Recovery of *miombo *woodland following charcoal production

The re-growth rate of *miombo *woodlands could not be determined during this two-year study. However, the resilience of *miombo *woodlands is a crucial question for the evaluation of the charcoal production system as a whole. Bailis [25] and Chidumayo [8,11,26] assume high recovery potentials for *miombo *woodlands and other woody savannas. Many of the species are re-sprouters - in other words, the rootstock is not killed when the aboveground biomass is harvested, and a vigorous coppice re-growth is soon developed [27]. It is assumed that this growth pattern predominates because elephants and fire have had significant impacts on *miombo *woodlands during their evolution [28]. The impacts of elephants and fire are comparable to charcoal production as long as a recovery period is permitted and seed survival is ensured [29]. If the woodland is allowed to regenerate to the pre-harvest biomass before being cut again, only the CH_4_, N_2_O, ozone precursors and aerosols produced during the combustion process represent 'additional' radiative forcing of the atmosphere in the decadal timeframe, since the CO_2 _is re-fixed during the recovery period.

However, it is questionable whether the observed rate of charcoal production is sustainable at a whole-country scale and how the energy demands of Zambia can be satisfied without woodland degradation. The minimum time for full aboveground biomass recovery in *miombo *woodlands after clear-felling is in the order of 30 years [8,9]. Therefore, the suggested rate of 1% forest clearance per year in Zambia [6,30] should theoretically be sustainable. The Zambian Urban Household Energy Study in 1988 came to the conclusion that 'the household sector can rely on wood fuel indefinitely if the woodland resource base is protected and properly managed' [cited as given in 11]. However, by our estimation, the current pressure on *miombo *woodlands for charcoal production is too high to allow sustainable usage, for two reasons:

• A significant portion of the woodlands initially cut for charcoal are subsequently converted to croplands, mostly for growing Cassava in our study area. Therefore the coppice-based recovery of the woodlands is prevented or delayed. Post-harvest usage for agriculture results in an extremely fast loss of soil organic matter [31], not observed where the aboveground tree biomass only is removed. The subsequent recovery rate of woodland is reduced when soil nutrients are depleted by cropping and recovery is primarily based on seedlings and not on strong, established rootstocks.

• The impact on forests is not evenly distributed. The charcoal production is concentrated around urban areas and along transport corridors [32,33]. This results in the development of zones of total deforestation around big settlements and along roads, as has happened around the capital, Lusaka, and another major city, Ndola, during the past decades. These losses are not compensated for by sustained sink behaviour or biomass increments in the less-impacted woodlands. Scholes and Biggs [34] found this to be generally true in Southern Africa: theoretical wood production rates exceed use rates at national scales, but not locally.

### Country-scale emissions from deforestation

The mean value of CO_2 _emissions from deforestation calculated in this study was 34.3 Mt CO_2 _y^-1 ^(within an uncertainty ranging from 13.2 Mt CO_2 _y^-1 ^to 102.8 Mt CO_2 _y^-1^). This value exceeds the estimation of 16 - 26.5 Mt CO_2 _y^-1 ^recently reported by UN REDD [7]. However, also the authors of the UN REDD [7] study express their discomfort about the low data availability and state that their estimates "should be considered only as indications." Further support for the Zambian Forestry Department to build up a forest monitoring system is highly desired.

Despite the high uncertainty it can be stressed that charcoal production is an extremely inefficient way to supply energy. If the calculations in our study were right, the annual emissions from deforestation in Zambia would be in the same order of magnitude as the biggest single CO_2 _emitter in the world - the coal-fired Taichung power plant at Taiwan^1 ^- that emitted 39.7 Mt CO_2 _y^-1 ^in 2008. However, even this carbon-intensive power plant provided three times more energy (39,800 GWh y^-1^) than the charcoal production in Zambia (11,700 GWh y^-1^). Notwithstanding, the Zambian population is highly depending on this inefficient energy source and as long as there are no alternatives the country will stay in a situation that we call the 'charcoal trap'.

## Conclusions

### How to escape the charcoal trap?

Action for escaping the charcoal trap can be taken at several levels:

• The pressure on *miombo *woodlands could be reduced by introducing charcoal kilns of higher efficiency [25] and improving the post-harvest management. Currently charcoal is produced in traditional earth kilns, which have an energy conversion efficiency of about 12%. More modern, but still simple designs, can achieve a three-fold increase in efficiency [35]. A side benefit is the reduced production of methane, non-methane hydrocarbons and aerosols. However, new technologies to improve efficiency of charcoal production very often meet social or traditional barriers that prevent implementation.

• Improved post-harvest management requires a stronger governmental and local (communal) land management regime, which in turn calls for socio-economic research on rural development. The charcoal production market system was analysed in depth during the 1990s [36] but more recent developments are not available. Recent forest inventories and realistic data on clearing rates, re-growth and turnover times are sparse.

• These efforts might reduce the degree of un-sustainability of the woodland usage, but in our opinion the long-term pressure on *miombo *woodlands can only be reduced significantly by a shift away from charcoal to other energy sources. Electrification could be a part of the solution, in a first step in fast growing urban areas, which currently have a high charcoal demand [4]. In a second step, electrification of rural areas should follow. Protection of natural woodland resources, biodiversity, and climate change mitigation are not the only arguments for rural electrification. Access to reliable and affordable electricity has further developmental benefits, for instance in health and education. It would also reduce the time and effort for collecting firewood [37]. Ilskog and Kjellström [38] recently described several economical alternatives to implement rural electrification projects. Gradually developing small local solutions in public-private-partnership may be the most promising solution. Haanyika [37] favours decentralised electricity supply systems such as mini-hydropower, biomass gasification and solar photovoltaics, which reduce the high cost associated with grid extension.

Integrating those different levels requires an integrated research and implementation concept that combines social, economic and environmental aspects. Further studies have to find out how the escape from the charcoal trap can be achieved and whether the UN REDD process can support this process, e.g. by providing finances for electrification and regenerative power generation.

Another key action is increasing the knowledge on forest regeneration. Currently, it is likely that Zambian forests are a net source of CO_2_, since they are over-harvested. However, the resilience of *miombo *woodlands is high and a thorough management could result in a sustainable source of regenerative energy provided by the forests. Better inventory data are urgently required to improve knowledge about the current state of the woodland usage and about recovery.

We conclude that net greenhouse gas emissions could be substantially reduced by post-harvest management, improvements in charcoal production technology, and alternative energy supply. A shift in energy supply from charcoal to electricity would reduce the pressure but requires high investments into grid and power generation. Since Zambia currently cannot generate this money by itself, the country is threatened to remain locked in the charcoal trap such as many other of its African neighbours. The question arises whether money and technology transfer to increase regenerative electrical power generation should become part of a post-Kyoto process.

## Methods

### Study site

The study was conducted in a *miombo *woodland within the Kataba Forest Reserve (15.43°S 23.25°E, 1084 m a.s.l.) in the Western Province of Zambia. The climate is tropical sub-humid with a distinct dry (May-mid October) and wet season (mid October - April). Annual long-term average air temperature is 21.8° C and the mean annual rainfall is 948 mm (Zambian Meteorological Department, Mongu Office). The soils are deep, nutrient-poor Kalahari sands. Kataba Forest Reserve was established by the Zambian government in 1973 to protect the indigenous vegetation. The woodland is characterised by a canopy cover of nearly 70% [39]. Dominant species are *Brachystegia spiciformis, Brachystegia bakerana, Guibourtia coleosperma *and *Ochna pulchra*, described by N'gok and Mosi [40] and Ernst [41] as characteristic species in *miombo *woodlands. Average canopy height is about 12 m. The surrounding areas have become highly disturbed by charcoal production and conversion to agricultural land, particularly over the past decade.

**Figure 3:**
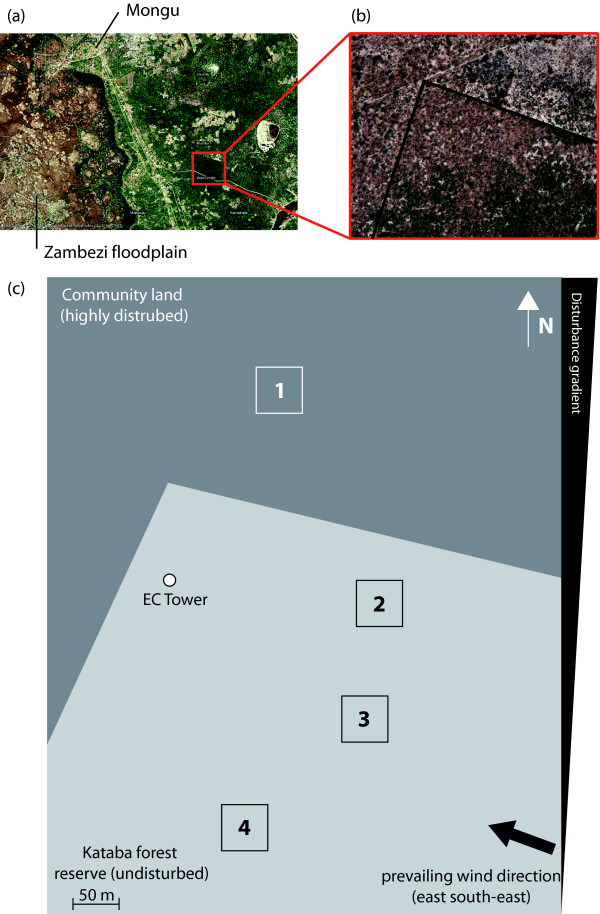
**Satellite pictures of the area and experimental design**.

The study comprised continuous eddy covariance measurements within the intact forest, combined with a series of inventory plots along a disturbance gradient (Figure 3) leading out into the surrounding area [16]. The lack in plot repetitions for the disturbed site has its reason in the project history: when the project was started the communal forest directly surrounding the reserve was almost intact, but it was cleared during first year of our measurements. In this situation we decided to add a fourth plot to the already existing three plots within the reserve. Given our resources during campaigns in this very remote area it was impossible to set up additional inventory plots. Since the heterogeneity within the reserve was very high compared to the cleared areas, we decided for this 3:1 design and tried to use our expert knowledge to find a representative plot in the cleared area.

### Eddy covariance measurements

Ecosystem-level fluxes of water, heat and carbon dioxide were measured using a closed-path eddy covariance system mounted at 19 meters above the ground on a tower. The system consisted of a 3-dimensional sonic anemometer (Solent R3, Gill Instruments, Lymington, UK) to measure fluctuations in horizontal and vertical wind speeds (m s^-1^) as well as the 'sonic temperature' (K); and a gas analyzer (LiCor 7000, LiCor, Lincoln, Nebraska, USA) to measure carbon dioxide (μmol mol^-1^) and water vapour (mmol mol^-1^) concentrations. The gas samples were drawn at about 5 l min^-1^ through a 4 m long teflon-coated tube to the gas analyser, which was enclosed in a weatherproof box. The sonic anemometer and gas analyser measurements were recorded at 20 Hz. The fluxes were calculated for 30 min intervals by means of the post-processing software package 'Eddysoft' [42]. The raw signals were converted into physical units and a planar fit rotation after Wilczak et al. [43] was applied. Time lags for CO^2^ and water vapour concentrations were calculated by determining the maximum correlation between the fluctuations of the concentrations and the vertical wind component w'. The fluxes were calculated using conventional equations [44-46]. The CO^2^ flux into and out of the air mass held within the vegetation-canopy storage was determined as the change over time of the CO^2^ profile between 0.1 and 18 m, where concentrations were measured continuously at 5 heights.

Data were filtered according to several quality criteria, including spikes in the raw data or the half-hourly data [47], stationarity [48], and situations with low or very high turbulence (u*-filtering)[49]. Gap-filling and flux partitioning was conducted by the standard procedures applied in the CarboAfrica database, described by Reichstein et al. [50]. Since the first rains commonly occur in October we set the start of the 'meteorological year' at Mongu to 1st September. Measurements were conducted from 15th September 2007 to 23^rd ^July 2009. The gaps in September 2007 and July and August 2009 were filled with corresponding data from the dry season in 2008.

About 60% of the time wind was blowing from the prevailing wind direction between 60° and 150°. In this sector, the terrain was totally flat and homogeneously covered by forest for at least 5 km. Data screening according Aubinet et al. [46] and Kutsch et al. [17] did not show any signs of advection when up-scaled process measurements were compared to night-time flux measurements [16].

### Inventory plots along the disturbance gradient

Three inventory plots (50 × 50 m) were set up in 2007 within the 50% fetch of the eddy covariance tower towards the prevailing wind direction, east-southeast [16]. The fourth plot was added during the first field campaign in March 2008. The four plots were then describing an inferred disturbance gradient, from the core of Kataba forest reserve towards the highly disturbed area north of the reserve (Figure 3). The following variables were recorded for each tree in each plot: species, height (m), diameter at breast height (dbh, cm) and base (dtb, cm), location in the plot (UTM coordinates) and damage class. The tree species encountered are listed in Table 4. We also determined leaf area index (LAI) by hemispheric photography, based on 100 photographs taken on a regular 5 × 5 m grid in each inventory plot using WinScanopy (Regent Instruments Inc., [51]).

**Table 4:** Tree species identified in the inventory plots

Scientific name	Losi name	Plot 1	Plot 2	Plot 3	Plot 4
*Brachystegia spiciformis*	mutuya	x	x	x	x
*Ochna pulchra*	munjelijeli	x	x	x	x
*Ekebergia capensis*	munyonga		x	x	x
*Burkea afrikana*	museshe		x	x	x
*Diospyros batoeana*	munjongolo		x	x	x
*Strychnos cocaloides*	muhuluhulu		x		
*Pterocarpus angolensis*	mukwa	x	x	x	x
*Guibourtia coleosperma*	muzaule	x	x	x	x
*Baphia obovata*	isunde	x	x	x	x
*Parinari curatellifolia*	mubula		x	x	x
*Diplorychos condylocarpon*	mulya	x	x	x	x
*Combretum psidioides*	mufula	x	x	x	
*Swartzia madagascariensis*	mushakashula		x		
*Vangneriopsis lanciflora*	mumousomouso				x
*Erythrophleum africanum*	mubako	x		x	x
*Brachystegia bakerana*	muluundu	x		x	
*Psydrax coleosperma*	munyanyo		x	x	

Standing aboveground biomass was calculated according to equations given in Nickless et al. [52]. Since it was impossible to derive a locally specific allometric equation (destructive sampling is prohibited in Kataba) we relied on a so-called broad-leafed allometric equation, which includes data on *Brachystegia spiciformis*, one of the key species of *miombo* woodlands. Similarily we derived the error terms as explained by Nickless et al. [52] and the values given represent a potential maximum of aboveground biomass in "old-growth" *miombo* woodlands. We could not rely on allometric equations given by Ryan et al. [10] for *miombo *woodlands in Mocambique, since *miombo *woodlands are highly diverse in canopy structure (e.g. with and without understory) and the *miombo *woodland under observation in Ryan et al. [10] is located at the dry end of *miombo*, characterized by trees smaller in size and diameter and an additional dense grass layer of about 1 m height.

For a detailed soil inventory an *a-priori *characterisation approach was conducted [16]. Each plot was divided into 100 subplots of 5 × 5 m and the ground cover in each subplot was characterised to find suitable homogeneous and representative patches. The postulated characterization of the soil heterogeneity was based on the abundance of ground cover types (mosses, grasses, litter, dead wood, bare ground etc). Each subplot was classified by its three most abundant types, e.g. LMF - litter, moss, free ground. The distribution of the sampling followed this phenomenological classification: for each category at least three samples were taken in every plot [16]). The chosen approach guarantees representativity of the various areas, accounting for rare areas being potential hot spots. Soil samples were collected in 2008 and 2009 (cores 4.8 cm in diameter, 30 cm in depth). The samples were air dried in the field and shipped to a laboratory in Jena, Germany and further analysed. For a more detailed description see Merbold et al. [16].

The impact of charcoal production was inferred from the aboveground biomass difference (*ΔB_a_*, t ha^-1^) between the three plots within the reserve (*B_a,undis_*, t ha^-1^) and the biomass remaining after charcoal production the disturbed site (*B_a,dis_*, t ha^-1^).

(1) $$\Delta B_{a}=B_{a,undis}-B_{a,dis}$$

### Energetical calculations

The biomass difference (*ΔB_a_*), average energy content of tropical hardwood (*ε_Wood_*, 15 GJ t^-1 ^for air dry wood[53]) and two conversion factors were used to calculate the energy supply provided by charcoal (Equation 2). Since fresh (wet) wood is used in charcoal production, the first factor describes the energy needed to evaporate the water in the wood and convert fresh wood into dry wood (c_dry _= 0.5, dimension-less, assuming a moisture content between 40 and 50% of the fresh weight^2^). The second factor describes the energy that is lost by chemically converting wood into charcoal (c_chem _= 0.3, dimension-less).

(2) $$E_{\sup}=\Delta B_{a}\times \varepsilon_{wood}\times c_{dry}\times c_{chem}$$

The supplied energy was calculated per ha of degraded miombo forest and was extrapolated to all of Zambia on the basis of reported deforestation rates.

### Upscaling

Estimations about the country-wide emissions as well as the gained energy require information about deforestation rates in Zambia and about the carbon pools in forests that underlie degradation or deforestation. Since this study was conducted in a protected area the observed biomass may be higher than the average biomass of *miombo *woodland. Therefore, we compiled additional information from literature sources and from recent assessments by the FAO and by UN-REDD [5-7,30]. However, the available information is sparse and highly uncertain. As a consequence, we produced a matrix of the different deforestation rates reported and the different stocks reported and presented ranges for countrywide emissions, per capita emissions and energy gained from charcoal production.

## Endnotes

^1 ^Taipeh Times, 4.9.2008, http://www.taipeitimes.com/News/biz/archives/2008/09/04/2003422196

^2 ^http://cta.ornl.gov/bedb/index.shtml

## Competing interests

The authors declare that they have no competing interests.

## Authors' contributions

WK and LM conceived this study and contributed equally to the manuscript, this includes writing process, experimental design and data analysis. WZ, MMM, MM and OK were involved in the experimental design and data collection. RJ Scholes contributed valuable input for the discussion section. All authors read and approved the final manuscript.
